# Effect assessment for the interaction between shaking table and eccentric load

**DOI:** 10.1038/s41598-022-19743-y

**Published:** 2022-09-12

**Authors:** Juke Wang, Aiwen Liu, Xiaojun Li, Zhenghua Zhou, Su Chen, Jinbao Ji

**Affiliations:** 1 Institute of Geophysics, China Earthquake Administration, Beijing, 100081 China; 2 Beijing Key Laboratory of Earthquake Engineering and Structural Retrofit, Beijing University of Technology, Beijing, 100124 China; 3 School of Transportation Engineering, Nanjing Tech University, Nanjing, 211816 China

**Keywords:** Civil engineering, Mechanical engineering

## Abstract

Electro-hydraulic shaking table is an essential experimental apparatus to evaluate structural performance under actual vibration condition. The control-structure interaction (CSI) between shaking table and eccentric load has lately received considerable attention for causing the accuracy degradation of shaking table test. At present, the research gap of the influence of the eccentricity of load on the CSI makes it challenging to find the CSI effects. And an effect assessment is yet to be proposed to evaluate the CSI effects, which has impeded the development of test technology. To overcome those theoretical bottlenecks, in this research, an analytical transfer function matrix of shaking table and eccentric load is established to analyze the CSI effects. The analysis is conducted under such conditions as different mass ratio (MR), moment of inertia ratio (IR), and eccentric distance ratio (ER) conditions. Through the analysis, the role of the ER is identified, the sensitivities of the MR, IR, and ER to the transfer function matrix are revealed, and the CSI effects are found. Furthermore, a novelty effect assessment is proposed to appraise whether the CSI effects can be ignored in shaking table test. And the visualization expression of the effect assessment is obtained for convenient application.

## Introduction

**Figure 1:**
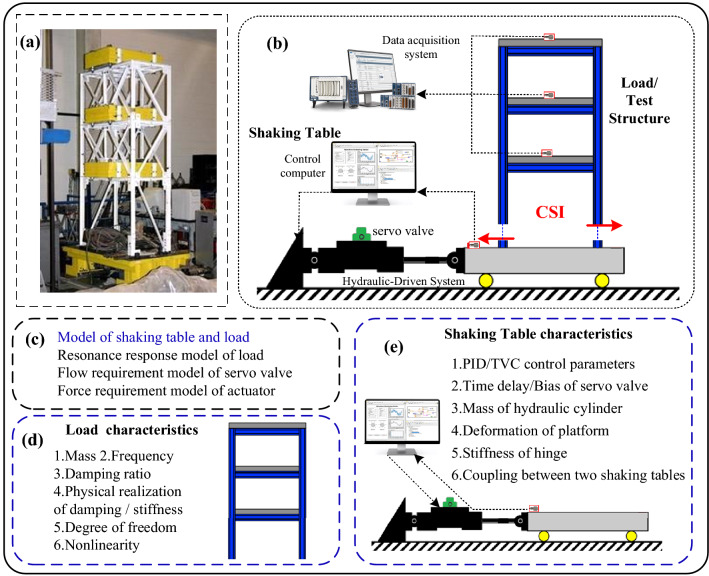
Analysis of the CSI effects. (**a**) Diagram of the shaking table and load, (**b**) dynamic coupling-CSI, (**c**) different Models, (**d**) different load characteristics, and (**e**) different shaking table characteristics.

Electro-hydraulic shaking table is one of the most important pieces of equipment for replicating actual vibration conditions in various applications, including biomedical and geological researches^1–3^, engineering seismic research works^4–6^, aerospace vibration tests^7,8^, and vehicle road simulations^9,10^, etc. In the context of shaking table test, the control-structure interaction (CSI)^11–13^ is the dynamic coupling between shaking table and load (that is the test structure model) (Fig. 1a,b). Due to the effects of the CSI, the control accuracy of shaking table and the accuracy of the response of load decrease to a great extent under some conditions^14–16^. Faced with these decreases, many researchers have taken shaking table and centrosymmetric load as research objects, and then established the models of shaking table and load (MST) and the other models (Fig. 1c). Based on the established models, the analysis of the CSI effects has been conducted with respect to two aspects: shaking table characteristics and structural (load) characteristics.

From the aspects of load characteristics, many researchers have analyzed the effects of mass, damping ratio, frequency rigidity and flexibility, the physical realization of damping and stiffness, degrees of freedom, and nonlinearity on the MST (Fig. 1d). Blondet and Esparza^17^ analyzed the CSI effects on the MST under different MR, structure damping ratio, and structure frequency. The analysis showed that at the frequency of structure and its surrounding frequency band, the CSI effects increases with the MR and structure frequency, and decreases with structure damping ratio. Wang et al.^18^ investigated the sensitivity of different test structure characteristics to the MST. The investigation revealed that structure frequency has the greatest influence on the MST, followed by structure damping ratio, and the MR is the smallest. Considering the dynamic coupling between base, platform, and structure, Conte and Trombetti^19,20^ conducted simulation analysis and experimental verification to analyze their effects on the MST. The research came to a conclusion that base has slight impact on the MST, but structure exerts significant influence on the MST. Furthermore, the research revealed that structure frequency becomes the second resonance frequency of whole system, and rigid load decreases the resonance peak of oil column frequency. According to the physical realization of damping and stiffness, Conte and Trombetti^20^ and Chen^21^ improved the modeling accuracy of shaking table and structure. The improvement proved that the construction mode of damping/stiffness have an effect on the MST. Huang^22^ studied the CSI effect between single degree of freedom structure and shaking table. The study demonstrated the MST has a resonance peak at the frequency of structure, and the resonance peak affects the control performance of shaking table. Tang et al.^23^ analyzed the CSI effects between shaking table and multi degree of freedom structure. The analysis revealed that the CSI exert effects on the MST at the previous natural frequency of structure. Guo et al.^24^ analyzed the CSI effects on shaking table tests. The analysis showed that the nonlinearity of test structure has great impact on the MST and the control accuracy of the shaking table decreases.

From the aspects of shaking table characteristics, many scholars have analyzed the effects of control parameters, the time delay and the bias of servo valve, the stiffness of hinge, the mass of hydraulic cylinder, the deformation of platform, and the coupling between two shaking tables on the MST (Fig. 1e). Trombetti et al.^20^ studied the sensitivities of control gain (PID control parameter, feedforward gain, and differential pressure control gain) and servo valve delay to the MST. The study revealed that the control gain and the delay of servo valve have significant impact on the MST. Li et al.^25^ analyzed the stability of the MST under different TVC parameters. The analysis showed that better system stability can be obtained with the no-load TVC control parameters. The influences of mechanical installation error, displacement measurement error, and bias of servo valve on the MST were considered in a study by Zhang^26^. The study shows the MST is only related to the bias of servo valve. And the CSI effects increases with the bias of servo valve. Yan studied the influence of the mass of exciter on the MST and found that the CSI effects increase with the mass of exciter^27^. The influence of the stiffness of hinge on the MST is investigated by Xie^28^. The investigation suggests that the stiffness hinge should be increased as much as possible. Maoult et al.^29^ adopted finite element modeling to investigate the phenomenon of structural frequency reduction in large-scale shaking table experiments. The investigation demonstrated that the deformation of shaking table is the main reason for the CSI effects. In other words, the deformation of shaking table is one of the factors that affect the MST. Wang^30^ and Li^31^ studied the CSI effect between dual shaking tables and structure. The study revealed that the coupling effect between the two shaking tables have significant impact on the MST at the frequency of structure and its surrounding frequency band.

Additionally, Wang et al.^32^ analyzed the CSI effects on the flow requirement model of servo valve, the force requirement model of exciter, and the resonance response model of load. The analysis was conducted under different load mass, the damping ratio of load, and the frequency of load conditions. The analysis shows the CSI must be considered in designing of the actuator force of actuator, and the flow requirement of servo valve can be achieved without considering the CSI when the flow requirement in the low-frequency band is satisfied.

To date, researchers have conducted studies that are limited to analyzing the CSI effects between shaking table and centrosymmetric load. However, most of loads in shaking table test are non-centrosymmetric. The CSI increases greatly when an eccentric load is loaded on shaking table in the studies of Zhao et al^14,33^. Guo et al.^34^ investigated the influence of different test structures on the control performance of shaking table, and the investigation showed that the control performance is greatly affected by the eccentricity of test structure. Therefore, it is necessary to study the CSI effects between shaking table and eccentric load. Moreover, the eccentric degree of load and the moment of inertia of load are important characteristics that have not been analyzed in previous studies. Furthermore, no effect assessment has been proposed to evaluate the CSI effects, and thus, the existing research needs to be further expanded.

In this study, an analytical transfer function matrix is established to analyze the CSI effects between twin-axes shaking table and eccentric load. Based on the transfer function matrix, an in-depth investigation is conducted under different MR, IR, and ER conditions. The sensitivities of the MR, the IR, and the ER to the transfer function matrix, as well as the influence frequency range and the influence trend of the CSI effects, are found in the investigation. Furthermore, a novelty effect assessment is proposed to appraise the CSI effects.

## Analytical modeling

**Figure 2:**
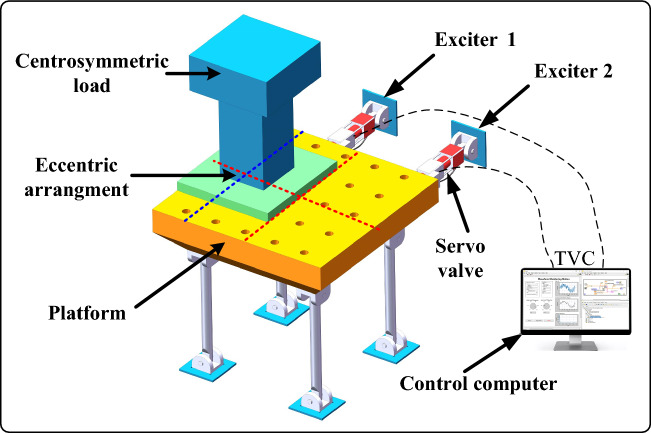
Schematic diagram of the shaking table and eccentric load.

The schematic diagram of the twin-axes shaking table and eccentric load is presented in Fig. 2. It can be seen from Fig. 2 that the shaking table is driven by two exciters in horizontal direction, and a centrosymmetric load is loaded on the platform eccentrically. It is worth noting that the ingenious arrangement can simulate the condition that an eccentric load is loaded on the platform to a great extent. Meanwhile, the analytical modeling process of the transfer function matrix is greatly simplified.

**Figure 3:**
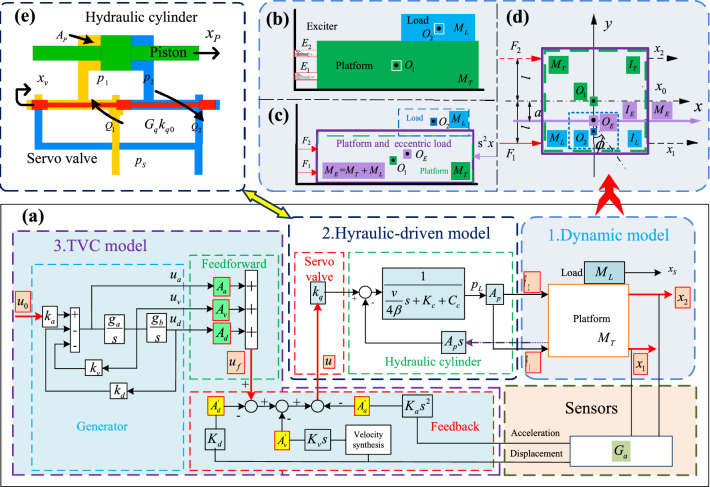
System modeling. (**a**) Three-sub models of the system, (**b**) dynamic model, (**c**) equivalent model of the dynamic model, (**d**) dynamic analysis of the equivalent model, and (**e**) hydraulic-driven model.

The transfer function matrix is established according to the modular method presented in Fig. 3. The method divides the shaking table and eccentric load into three main sub-models, including the dynamic model, the hydraulic-driven model, and the TVC model, which are shown in Fig. 3a. It should be noted that the components and the physical characteristics of the three sub-models are comprehensively considered in the modeling process. Detailed explanations of symbols are given in the following description of the modeling process, and the values of the parameters are listed in Table S1 in the Supplementary Materials.

### Dynamic system modeling

The dynamic model is the motion mechanism part of the shaking table and eccentric load. Without regard to the stiffness and the damping between the platform and the connecting rod, the dynamic model of the shaking table and eccentric load is shown in Fig. 3b–d. The detailed explanation of symbols is given as follows. $M_{T}$ is the mass of the platform, $M_{L}$ is the mass of the load, $E_{1}$ is exciter 1 of the shaking table, $E_{2}$ is exciter 2 of the shaking table, $O_{1}$ is the center of gravity of the platform, $O_{2}$ is the center of gravity of load, $F_{1}$ is the exciter force of exciter 1, $F_{2}$ is the exciter force of exciter 2, $x_{1}$ is the displacement of exciter 1, $x_{2}$ is the displacement of exciter 2, $I_{T}$ is the moment of inertia of the platform relative to its own centroid axis, $I_{L}$ is the moment of inertia of the load relative to its own centroid axis, $M_{E}$ is the equivalent mass of the platform and load, $I_{E}$ is the equivalent moment of inertia of the platform and load relative to its own centroid axis, $O_{E}$ is the center of gravity of equivalent mass, $x$ is the displacement of the equivalent mass, and $\phi$ is the motion angle of equivalent moment of inertia, $a$ is the distance from the center of gravity of the equivalent mass to the center of gravity of shaking table, and $l$ is the distance from exciter to the center of gravity of shaking table.

Figure 3c presents the dynamic model of the shaking table and eccentric load. In Fig. 3d, the shaking table and eccentric load are regarded as an integrated whole, which is an equivalent model of the dynamic model. Figure 3e depicts the analysis of the mechanical properties of the equivalent model. Based on the parallel axis theorem, the equivalent model can be expressed as follows:

1 $$\left. \begin{gathered} M_{E} = M_{T} + M_{L} \hfill \\ I_{E} = I_{T} + I_{TA} + I_{L} + I_{LA} \hfill \\ \end{gathered} \right\},$$

where $I_{T} { + }I_{TA}$ is the moment of inertia of the shaking table relative to the $x$-axis in Fig. 3d, $I_{L} { + }I_{LA}$ is the moment of inertia of the load relative to the $x$-axis in Fig. 3d. The calculation method of the moment of inertias is given detailly in the Supplementary Materials.

Based on Eq. (1), the Newton’s second law is adopted to establish the dynamic model of the shaking table and eccentric load. Then the dynamic model is

2 $$\left. \begin{gathered} M_{E} s^{2} x = F_{1} + F_{2} \hfill \\ I_{E} s^{2} \phi = F_{1} (l - a) - F_{2} (l + a) \hfill \\ \end{gathered} \right\}.$$

### Hydraulic-driven system modeling

The hydraulic-driven system is the driven part of the shaking table and eccentric load. The servo valve and the hydraulic cylinder are the two core components of hydraulic-driven system, the schematic diagram of which is demonstrated in Fig. 3e.

#### Servo valve

Generally, servo valve can be regarded as a second-order oscillation link^35^, and the transfer function of servo valve is

3 $$k_{q} = G_{q} k_{q0} = \frac{{k_{q0} }}{{\frac{{s^{2} }}{{n_{q}^{2} }} + \frac{{2D_{q} }}{{n_{q} }}s + 1}},$$

where $k_{q0}$ is the flow gain of servo valve, $n_{q}$ is the frequency of servo valve, and $D_{q}$ is the damping ratio of servo valve.

#### Hydraulic cylinder

Combined with servo valve and hydraulic cylinder, the hydraulic-driven system can be expressed by a series of continuity equations^36^. The Laplace transform is applied to the continuity equations, and the converted form can be described as follows

4 $$\left. \begin{gathered} Q_{L} = G_{q} k_{{q{0}}} u - K_{C} p_{L} \hfill \\ Q_{L} = A_{p} sx_{T} + \frac{V}{4\beta }sp_{L} \hfill \\ A_{p} p_{L} = M_{TL} s^{2} x_{T} \hfill \\ \end{gathered} \right\},$$

where $Q_{L}$ is the flow of servo valve, $G_{q} k_{q0}$ is the transfer function of servo valve, $u$ is the control error signal, $p_{L}$ is the load pressure, $A_{P}$ is the effective area of cylinder, $x_{T}$ is the displacement of the shaking table, $V$ is the total capacity of two hydraulic cylinder chambers, $\beta$ is effective bulk modulus, and $M_{TL}$ is the total mass of load, platform and exciter piston.

### TVC system modeling

The TVC model is the control part of the shaking table and eccentric load. The TVC model consists of feedback loop, feedforward loop, and generator of the TVC, the schematic diagram of which is presented in Fig. 3a.

According to the TVC system modeling process presented in Ref.^32^, the control error signal is

5 $$u = G_{3} u_{0} - G_{4} G_{a} x_{T} ,$$

where $u$ is control error signal, $u_{0}$ is control signal, $G_{3}$ is the generator and the feedforward of the TVC, $G_{4}$ is the feedback of the TVC, $x_{T}$ is the displacement of shaking table, and $G_{a}$ is the transfer function of sensors. The analytical expression of $G_{a}$ is

6 $$G_{a} = \frac{1}{{\frac{{s^{2} }}{{n_{a}^{2} }} + \frac{{2D_{a} s}}{{n_{a} }} + 1}},$$

where $n_{a}$ is the frequency of sensors and $D_{a}$ is the damping ratio of sensors.

### Transfer function matrix modeling

The analytical transfer function matrix is established based on the three sub-models. Assuming the parameters of exciter 1 and 2 are the same, the transfer function matrix can be described as

7 $$\left. \begin{gathered} \left\{ {\frac{{G_{2} }}{{4l^{2} }}[M_{E} s^{2} (l + a)^{2} + I_{E} s^{2} ] + s} \right\}x_{1} + \left\{ {\frac{{G_{2} }}{{4l^{2} }}[M_{E} s^{2} (l^{2} - a^{2} ) - I_{E} s^{2} ]} \right\}x_{2} = \frac{{G_{q} k_{{q{0}}} u_{1} }}{{A_{p} }} \hfill \\ \left\{ {\frac{{G_{2} }}{{4l^{2} }}[M_{E} s^{2} (l - a)^{2} + I_{E} s^{2} ] + s} \right\}x_{2} + \left\{ {\frac{{G_{2} }}{{4l^{2} }}[M_{E} s^{2} (l^{2} - a^{2} ) - I_{E} s^{2} ]} \right\}x_{1} = \frac{{G_{q} k_{{q{0}}} u_{2} }}{{A_{p} }} \hfill \\ \end{gathered} \right\},$$

where $u_{1}$ and $u_{{2}}$ are the control error signals of two exciters, $G_{q} k_{{q{0}}}$ is the transfer function of servo valve, and the expression of $G_{{2}}$ is

8 $$G_{2} = \frac{V}{{4\beta A_{P}^{2} }}s + \frac{{K_{C} + C_{C} }}{{A_{p}^{2} }}.$$

Substituting Eqs. (5), (6) and (8) into Eq. (7), the analytical transfer function matrix can be obtained as follows:

9 $$\left. \begin{gathered} \left\{ {\frac{{G_{2} }}{{4l^{2} }}[M_{E} s^{2} (l + a)^{2} + I_{E} s^{2} ] + s + \frac{{G_{q} k_{q} }}{{A_{p} }}G_{4} G_{a} } \right\}x_{1} + \left\{ {\frac{{G_{2} }}{{4l^{2} }}[M_{E} s^{2} (l^{2} - a^{2} ) - I_{E} s^{2} ]} \right\}x_{2} = \frac{{G_{3} G_{q} k_{q} u_{1} }}{{A_{p} }} \hfill \\ \left\{ {\frac{{G_{2} }}{{4l^{2} }}[M_{E} s^{2} (l - a)^{2} + J_{E} s^{2} ] + s + \frac{{G_{q} k_{q} }}{{A_{p} }}G_{4} G_{a} } \right\}x_{2} + \left\{ {\frac{{G_{2} }}{{4l^{2} }}[M_{E} s^{2} (l^{2} - a^{2} ) - J_{E} s^{2} ]} \right\}x_{1} = \frac{{G_{3} G_{q} k_{q} u_{2} }}{{A_{p} }} \hfill \\ \end{gathered} \right\}.$$

Converting Eq. (9) into a transfer function matrix as follows:

10 $$\left\{ \begin{gathered} x_{1} \hfill \\ x_{2} \hfill \\ \end{gathered} \right\} = \left[ {\begin{array}{*{20}c} {H_{11} } & {H_{12} } \\ {H_{21} } & {H_{{2{2}}} } \\ \end{array} } \right]\left\{ \begin{gathered} u_{1} \hfill \\ u_{2} \hfill \\ \end{gathered} \right\},$$

where $H_{11}$ and $H_{{2{2}}}$ are the transfer function of two exciters which are affected by the CSI, $H_{{1{2}}}$ and $H_{21}$ are the transfer functions of the coupling between two exciters, and $u_{1}$ and $u_{{2}}$ are the control signals of two exciters. The expressions of $H_{11}$, $H_{{1{2}}}$, $H_{21}$ and $H_{{2{2}}}$ are

11 $$\left. \begin{gathered} H_{11} = \frac{{G_{3} G_{q} k_{q} }}{{A_{p} }}\frac{{G_{7} }}{{G_{5} G_{7} - G_{6}^{2} }} \hfill \\ H_{12} = H_{21} = \frac{{G_{3} G_{q} k_{q} }}{{A_{p} }}\frac{{ - G_{6} }}{{G_{5}^{2} - G_{6}^{2} }} \hfill \\ H_{22} = \frac{{G_{3} G_{q} k_{q} }}{{A_{p} }}\frac{{G_{5} }}{{G_{5} G_{7} - G_{6}^{2} }} \hfill \\ \end{gathered} \right\},$$

where $G_{3}$ is the transfer function of the generator and the feedforward of the TVC. $G_{{5}}$, $G_{{6}}$, and $G_{{7}}$ can be written as

12 $$\left. \begin{gathered} G_{5} = \frac{{G_{2} }}{{4l^{2} }}[M_{E} s^{2} (l + a)^{2} + I_{E} s^{2} ] + s + \frac{{G_{q} k_{q} }}{{A_{p} }}G_{4} G_{a} \hfill \\ G_{6} = \frac{{G_{2} }}{{4l^{2} }}[M_{E} s^{2} (l^{2} - a^{2} ) - I_{E} s^{2} ] \hfill \\ G_{7} = \frac{{G_{2} }}{{4l^{2} }}[M_{E} s^{2} (l - a)^{2} + I_{E} s^{2} ] + s + \frac{{G_{q} k_{q} }}{{A_{p} }}G_{4} G_{a} \hfill \\ \end{gathered} \right\},$$

where $G_{{4}}$ is the transfer function of the feedback of the TVC, and $G_{a}$ is the transfer function of sensors.

## Analysis of the CSI effects

Based on the transfer function matrix, an in-depth analysis is conducted to find the sensitivity of the MR, IR, and ER to the transfer function matrix, and the influence trend and degree of the CSI effects on the transfer function matrix. The analysis is carried out under different MR, IR, and ER conditions. According to the Tables S2 and S3 in the Supplementary Materials, the different MR, IR, and ER conditions are presented in Table 1. In Table 1, the MR is denoted by ${\text{MR = }}M_{L} {/}M_{T}$, the IR is denoted by ${\text{IR = }}I_{L} {/}I_{T}$, and the ER is denoted by ${\text{ER}} = a{/}l$ ($l$ is 1.2 m).

**Table 1:** Different MR, IR, and ER conditions.

Conditions	MR	IR	ER
Different MR conditions	MR = 0.5	IR = 0.10	ER = 0.4
	MR = 1.0		
	MR = 1.5		
Different IR conditions	MR = 1.0	IR = 0.05	ER = 0.4
		IR = 0.10	
		IR = 0.15	
Different ER conditions	MR = 1.0	IR = 0.10	ER = 0.2
			ER = 0.4
			ER = 0.6

For comparing and analyzing the CSI effects on the transfer function matrix, the condition that the shaking table with no load is defined as a reference condition. In the analysis, the absolute value of the amplitude frequency characteristics of $H_{11}$ and $H_{22}$ is taken as a quantization index. If the quantization index exceeds ± 3.00 dB, it means that the shaking table will not work in its working frequency range.

**Figure 4:**
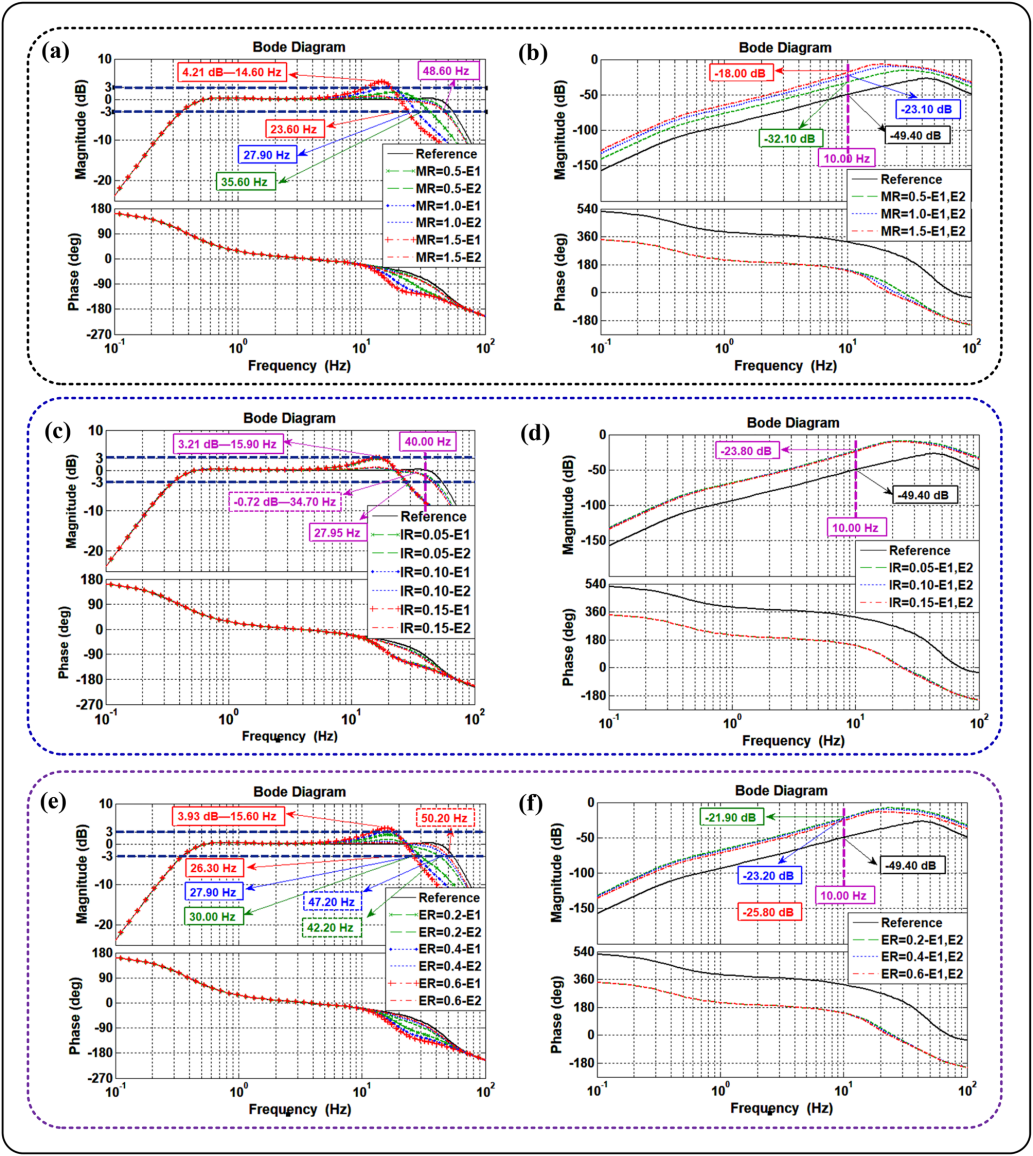
CSI effects on the transfer function matrix. (**a**) Effects of MR on $H_{11}$ and $H_{22}$, (**b**) effects of MR on $H_{12}$ and $H_{21}$, (**c**) effects of IR on $H_{11}$ and $H_{22}$, (**d**) effects of IR on $H_{12}$ and $H_{21}$, (**e**) effects of ER on $H_{11}$ and $H_{22}$, and (**f**) effects of ER on $H_{12}$ and $H_{21}$ (E1 and E2 are the two exciters of shaking table).

Figure 4 presents the CSI effect on the transfer function matrix under different MR, IR, and ER conditions. Figure 4a shows that at 35.60 Hz the value of $H_{11}$ is − 3.00 dB when MR = 0.50, at 27.90 Hz the value of $H_{11}$ is − 3.00 dB when MR = 1.0, and at 23.60 Hz the value of $H_{11}$ is − 3.00 dB when MR = 1.50. At the same time, with the changes of the MR (from 0.5 to 1.5), the value of $H_{22}$ remains to be − 3.00 dB at 48.60 Hz. Based on the above data, it can be obtained that at the resonance peak of oil column frequency and its surrounding frequency band, the influence of the CSI on $H_{11}$ increases with the MR, and the MR has no obvious effect on $H_{22}$. Besides, the frequency of the resonance peak of oil column of $H_{11}$ decreases with the MR, and the value of the resonance peak of oil column of $H_{11}$ increases with the MR. It can be observed from Fig. 4b that at 10.00 Hz, the value of $H_{{1{2}}}$ and $H_{{{21}}}$ is − 49.40 dB under the reference condition, but it is − 32.10 dB when MR = 0.5, − 23.10 dB when MR = 1.0, and − 18.00 dB when MR = 1.5. According to these collected data, it can be concluded that with the increase of the MR, the coupling between two exciters increases rapidly. The coupling is amplified at least 31.40 dB (about 37.15 times).

Figure 4c shows that although the value of the IR changes, the values of $H_{11}$ are − 3.00 dB at 27.95 Hz and 3.21 dB at 15.90 Hz, and the values of $H_{22}$ are − 0.72 dB at 34.70 Hz. Figure 4d illustrates the fact that although the IR conditions are different, both of the values of $H_{12}$ and $H_{21}$ are 23.80 dB at 10.00 Hz. Based on the above data, it can be drawn that the IR only has a slight effect (the quantization index does not exceed ± 1.00 dB) on $H_{12}$ and $H_{21}$ when the frequency exceeds 40.00 Hz. The sensitivity of the IR to each transfer function in the transfer function matrix is low.

Figure 4e shows that at 30.00 Hz, the value of $H_{11}$ is − 3.00 dB when ER = 0.2, at 27.90 Hz the value of $H_{11}$ is − 3.00 dB when ER = 0.4, at 26.30 Hz the value of $H_{11}$ is − 3.00 dB when ER = 0.6, and at 15.60 Hz the value of $H_{11}$ is 3.93 dB when ER = 0.6. Meanwhile, at 42.20 Hz the value of $H_{{{22}}}$ is − 3.00 dB when ER = 0.2, at 47.20 Hz the value of $H_{{{22}}}$ is − 3.00 dB when ER = 0.4, and at 50.20 Hz the value of $H_{{{22}}}$ is − 3.00 dB when ER = 0.6. According to the data collected, it can be obtained that at the resonance peak of oil column frequency and its surrounding frequency band, the influence of the CSI on $H_{11}$ increases with the ER, and the influence of the CSI on $H_{22}$ decreases with the ER. Meanwhile, the frequency of the resonance peak of oil column of $H_{11}$ decreases with the ER, and the value of the resonance peak of oil column of $H_{11}$ increases with the ER. Besides, the effective frequency band of $H_{{{22}}}$ increases at a certain extent, but the frequency does not exceed the effective frequency band of the reference condition. It can be observed from Fig. 4f that at 10.00 Hz, the values of both $H_{12}$ and $H_{21}$ are − 21.90 dB when ER = 0.2, − 23.20 dB when ER = 0.4, and − 25.80 dB when ER = 0.6. These data show that the coupling between the two exciters decreases with the ER.

According to Fig. 4, the MR, ER, and IR have no evident effect on the phase diagram of the transfer function matrix (the quantization index does not exceed ± 60°), and thus, the phase diagram is not discussed in this paper. Besides, the role of the ER can be identified as follows. The ER exerts effects on the transfer function matrix at the resonance peak of oil column frequency and its surrounding frequency band.

## Assessment of the CSI effects

The above “Analysis of the CSI effects” proves that the CSI exerts different degrees of effects on the transfer function matrix. However, the above analysis needs to be further expanded to appraise the CSI effect in practical experiments. To appraise whether the CSI effects can be ignored in shaking table test, a novelty effect assessment is proposed. Two steps are involved in proposing the assessment. The first step is the deduction of the assessment, and the second step is the visualization expression of the assessment.

### Deduction of the effect assessment

The “Analysis of the CSI effects” proves that the MR, IR, and ER have influence on the $H_{11}$ and $H_{22}$. In the light of Eq. (1) and the parallel axis theorem, the MR, IR, and ER are related to the mass and the inertia moment of the system. The relationship indicates that the CSI effects are related to two physical quantities, the moment of inertia and the mass of the system. In the deduction of the effect assessment, the ratio of the moment of inertia of shaking table to the moment of inertia of the system (IRS) and the MR are adopted to represent the two physical quantities.

It is assumed that the assessment can be expressed as follows:

13 $$CSI(IRS) = f_{1} \left( {IR,ER,MR} \right),$$

14 $$CSI(MR) = f_{2} \left( {MR} \right),$$

where $IRS$ is the ratio of the $I_{T}$ to the $I_{E}$, and the expression of $IRS$ is

15 $$IRS = \frac{{I_{T} }}{{I_{E} }} = \frac{{I_{T} }}{{I_{T} + I_{TA} + I_{L} + I_{LA} }}.$$

Referring to the expressions of $I_{T}$, $I_{TA}$, $I_{L}$, and $I_{LA}$ in Supplementary Materials, Eq. (13) can be written as

16 $$CSI(IRS) = f_{1} \left( {\frac{{(m^{2} + n^{2} ) \cdot M_{T} }}{\begin{gathered} (m^{2} + n^{2} ) \cdot M_{T} + {12} \cdot M_{T} \cdot (\frac{{M_{L} }}{{M_{T} + M_{L} }}A)^{2} \hfill \\ + (c^{2} + k^{2} ) \cdot M_{L} + {12} \cdot M_{L} \cdot (\frac{{M_{T} }}{{M_{T} + M_{L} }}A)^{2} \hfill \\ \end{gathered} }} \right),$$

where $m$ and $n$ are the length and the width of the shaking table, respectively. $c$ and $k$ are the length and the width of the load, respectively. And $A$ is the distance from the center of gravity of the load to the center line of shaking table.

Employing $MR = M_{L} /M_{T}$, Eq. (16) can be expressed more compactly as

17 $$CSI(IRS) = f_{1} \left( {\frac{{m^{2} + n^{2} }}{\begin{gathered} m^{2} + n^{2} + (c^{2} + k^{2} ) \cdot MR \hfill \\ + {12} \cdot a^{2} \cdot MR/(1 + MR) \hfill \\ \end{gathered} }} \right).$$

According to $ER = a/l$, Eq. (17) can be conveniently transformed into

18 $$CSI(IRS) = f_{1} \left( {\frac{{m^{2} + n^{2} }}{\begin{gathered} m^{2} + n^{2} + (c^{2} + k^{2} ) \cdot MR \hfill \\ + {12} \cdot ER^{2} \cdot l^{2} \cdot MR/(1 + MR) \hfill \\ \end{gathered} }} \right).$$

Finally, Eq. (13) can be obtained as follows:

19 $$CSI(IRS) = f_{1} \left( {\frac{{1}}{{{1} + IR + 12 \cdot ER^{2} \cdot \frac{{l^{2} }}{{(m^{2} + n^{2} )}} \cdot \frac{MR}{{1 + MR}}}}} \right).$$

With the combination of Eq. (14), the assessment can be described as follows:

20 $$\left. \begin{gathered} CSI(IRS) = f_{1} \left( {\frac{{1}}{{{1} + IR + 12 \cdot ER^{2} \cdot \frac{{l^{2} }}{{(m^{2} + n^{2} )}} \cdot \frac{MR}{{1 + MR}}}}} \right) \hfill \\ CSI(MR) = f_{2} \left( {MR} \right) \hfill \\ \end{gathered} \right\}.$$

### Visualization expression of the effect assessment

For the convenient application of the assessment, Eq. (20) is simplified and visualized as follows. According to the conclusion in “Analysis of the CSI effects” that the changes of the IR have slight effect on the transfer function matrix. If the IR is set to be 0.1 (the value of IR is determined according to the characteristics of load in Table S2 in the Supplementary Materials), then Eq. (20) can be simplified as

21 $$\left. \begin{gathered} CSI(IRS) = f_{1} \left( {\frac{{1}}{{{1}{\text{.1}} + 12 \cdot ER^{2} \cdot \frac{{l^{2} }}{{(m^{2} + n^{2} )}} \cdot \frac{MR}{{1 + MR}}}}} \right) \hfill \\ CSI(MR) = f_{2} \left( {MR} \right) \hfill \\ \end{gathered} \right\}.$$

Referring to the basic characteristics of the shaking table in Table S3 in the Supplementary Materials, then Eq. (21) can be expressed as

22 $$\left. \begin{gathered} CSI(IRS) = f_{1} \left( {\frac{{1}}{{{1}{\text{.1}} + 0.96 \cdot ER^{2} \cdot \frac{MR}{{1 + MR}}}}} \right) \hfill \\ CSI(MR) = f_{2} \left( {MR} \right) \hfill \\ \end{gathered} \right\}.$$

It can be analyzed from Eq. (22) that the CSI effects are related to the MR and ER. Based on the analysis, a visualization expression of the assessment can be obtained. The concept for obtaining the visualization expression is presented as follows. Firstly, to appraise whether the CSI effects can be ignored, a judgment standard needs to be proposed. The standard is that if the amplitude characteristics of $H_{11}$ and $H_{22}$ exceed the range of ± 3.00 dB or the effective frequency band of $H_{11}$ and $H_{22}$ decreases to 70% of the frequency band of the reference condition, the CSI effects cannot be ignored. Secondly, the values of the MR and ER are determined according to the standard. In the determination process, the range of the MR is investigated under the assumption of ER = 0, and then the ER is obtained under specific value of MR.

**Figure 5:**
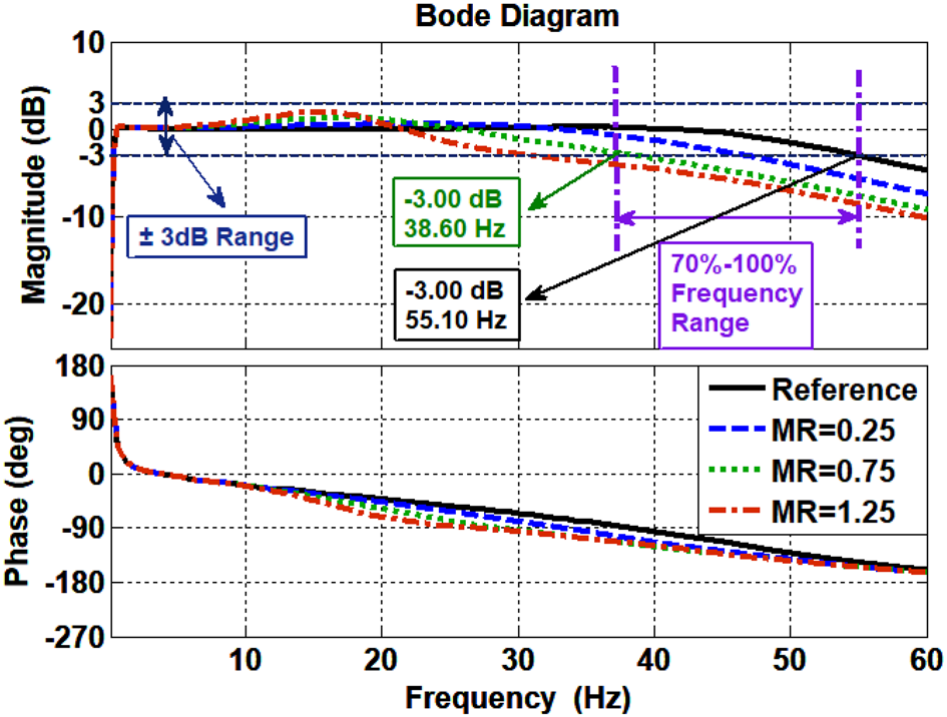
Determination of the MR.

According to the concept, the characteristics of the $H_{11}$ and $H_{22}$ under different MR are determined. The result of the determination is presented in Fig. 5. It can be seen from Fig. 5 that when MR = 0.75 the values of $H_{11}$ and $H_{22}$ are both − 3.00 dB at 38.60 Hz, which just meets the judgment standard. It can be concluded that the MR ≤ 0.75.

**Figure 6:**
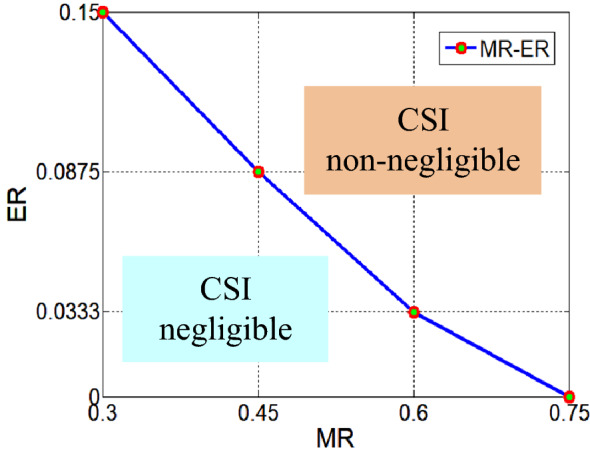
Visualization expression of the assessment.

Based on the conclusion that the MR ≤ 0.75, the specific values of MR, − 0.75, 0.60, 0.45, and 0.30, are adopted to obtain the values of the ER. Then the coordinate points (${\text{MR}}$, ${\text{ER}}$) are drawn in rectangular coordinate system, and a curve can be obtained by connecting the points (MR, ER). The curve is the visualization expression of the assessment of which is shown Fig. 6. To illustrate the specific meaning of the coordinate points, the point (0.45, 0.0875) is taken as an example. The point (0.45, 0.0875) indicates that when MR = 0.45, the CSI effects can be ignored if ER ≤ 0.0875. If a point (MR, ER) is above the curve, the CSI cannot be ignored, and if a point (MR, ER) is below the curve, then the CSI can be ignored.

## Conclusions

In this study, an analytical transfer function matrix is established to analyze the CSI effects between the twin-axes shaking table and eccentric load. Based on the transfer function matrix, a comprehensive investigation is conducted under different ${\text{MR}}$, ${\text{IR}}$, and ${\text{ER}}$ conditions. The sensitivities of the ${\text{MR}}$, ${\text{IR}}$, and ${\text{ER}}$ to the transfer function matrix, the influence frequency range and the influence trend of the CSI effects are obtained. Furthermore, a novelty effect assessment is proposed to appraise whether the CSI effects can be ignored in shaking table test. The most important conclusions can be drawn as follows.

1. The ER exerts effects on the transfer function matrix at the resonance peak of oil column frequency and its surrounding frequency band.
2. The transfer function matrix is the most sensitive to the changes of the MR, followed by the ER and then the IR. The IR has slight influence on the transfer function matrix. Additionally, the CSI exerts different influence trend and the influence degree on the transfer function matrix under different MR, ER, and IR conditions.
3. The CSI leads to a significant increase in the exciter coupling. The coupling between two exciters is amplified at least 37.15 times.
4. The novelty effect assessment has a wide application prospect and practical value in appraising whether the CSI effects can be ignored in shaking table test.
5. It is worth expanding the assessment to appraise the CSI effect between shaking table and flexible structure in the future.

## Supplementary Information

Supplementary Information.

## Data Availability

All data is available in the main text or the Supplementary Information. The data are available from the corresponding author upon reasonable request.
