# Supplementary material for: Effect assessment for the interaction between shaking table and eccentric load
Source: Sci Rep. 2022 Sep 12;12:15349. doi: 10.1038/s41598-022-19743-y (PMC9468167; doi:10.1038/s41598-022-19743-y)
Supplement: Supplementary file 1 — Supplementary Information. [file 41598_2022_19743_MOESM1_ESM.docx]

**Supplementary Materials**

**Effect assessment for the interaction between shaking table and eccentric load**

# Juke Wang ^1^, Aiwen Liu ^1,+^, Xiaojun Li ^2^,*, Zhenghua Zhou ^3,+^, Su Chen ^2,+^, and Jinbao Ji ^2^

^1^China Earthquake Administration, Institute of Geophysics, Beijing, 100081, China

^2^Beijing University of Technology, Beijing Key Laboratory of Earthquake Engineering and Structural Retrofit, Beijing, 100124, China

^3^Nanjing Tech University, School of Transportation Engineering, Nanjing, 211816, China

^*^Corresponding author: Xiaojun Li, beerli@vip.sina.com

^+^these authors contributed equally to this work

# Parameters of the shaking table and load

The values of the parameters of shaking table are listed in Table S1, the basic characteristics of load under different conditions are presented in Table S2, and the performance indexes of the shaking table are given in Table S3.

**Table S1.** Parameters of the shaking table.

| Parameters | Values | Parameters | Values |
| --- | --- | --- | --- |
| $A_{P}$ | $1.1\times10^{-2}\text{ m}^{2}$ | $k_{q0}$ | $1.364\times10^{-2}\text{ m}^{3}\text{/s}\cdot\text{V}$ |
| $\beta$ | $7\times10^{8}\text{ N/m}^{2}$ | $V$ | $2.662\times10^{-3}\text{ m}^{3}$ |
| $K_{C}$ | $2.5\times10^{-11}\text{ m}^{5}\text{/N/s}$ | $n_{q}$ | $628\text{ rad/s}$ |
| $D_{q}$ | $0.7$ | $n_{a}$ | $942\text{ rad/s}$ |
| $D_{a}$ | $0.7$ | $K_{d}$ | $100\text{ V/m}$ |
| $K_{v}$ | $\text{16.67 V}\cdot\text{s/m}$ | $K_{a}$ | $\text{0.5 V}\cdot\text{s}^{2}\text{/m}$ |
| $g_{a}$ | $31.25$ | $g_{b}$ | $6.4$ |
| $k_{a}$ | $2$ | $k_{v}$ | $0.0965$ |
| $k_{d}$ | $0.0202$ | $A_{d}=A_{d}^{'}$ | $0.19$ |
| $A_{\nu}$ | $2.8\times10^{-2}$ | $A_{\nu}^{'}$ | $-0.012$ |
| $A_{a}$ | $3.6\times10^{-3}$ | $A_{a}^{'}$ | $4.6\times10^{-3}$ |

**Table S2.** Basic characteristics of load under different conditions.

| Different  Loads | Mass  ($\text{kg}$) | Moment of inertia  ($\text{kg}\times\text{m}^{2}$) | Eccentric distance ($\text{m}$) | Density ($\text{kg/m}^{3}$) | Length, width, and height ($\text{m}\times\text{m}\times\text{m}$) |
| --- | --- | --- | --- | --- | --- |
| 1 | 3000 | 900 | 0.48 | 2500 | 1.34×1.34×0.67 |
| 2 | 6000 | 900 | 0.48 | 5000 | 0.95×0.95×1.33 |
| 3 | 9000 | 900 | 0.48 | 7500 | 0.78×0.78×2.00 |
| 4 | 6000 | 450 | 0.48 | 7500 | 0.67×0.67×1.78 |
| 5 | 6000 | 900 | 0.48 | 5000 | 0.95×0.95×1.33 |
| 6 | 6000 | 1350 | 0.48 | 2500 | 1.16×1.16×1.78 |
| 7 | 6000 | 900 | 0.24 | 5000 | 0.95×0.95×1.33 |
| 8 | 6000 | 900 | 0.48 | 5000 | 0.95×0.95×1.33 |
| 9 | 6000 | 900 | 0.72 | 5000 | 0.95×0.95×1.33 |

**Table S3.** Performance indexes of the shaking table.

| Parameters | Values |
| --- | --- |
| Platform size | 3.0 m × 3.0 m |
| Platform mass | 6000 kg |
| Moment of inertia of platform | $\text{9000 kg}\times\text{m}^{2}$ |
| Driving type/Control mode | Electro-hydraulic servo control/Acceleration |
| The range of working frequency | 0.4-50 Hz |
| Exciter stroke | ± 100 mm |
| Maximum velocity | 0.6 m/s |
| Maximum acceleration | Non-loaded, 2.0 g and fully loaded, 0.9 g |
| Maximum load | 10,000 kg |

# Calculation of the moment of inertias

According to the parameters of the shaking table and the load listed in Table S2 and Table S3, the moment of inertias in different load conditions of Table 1 can be calculated by the following formula

|  | $I_{E}=I_{T}+I_{TA}+I_{L}+I_{LA}$ | (S1) |
| --- | --- | --- |

Taking the center of the shaking table as the zero point of the coordinate, and adopting the formula of centroid to calculate the centroid location, the centroid of the shaking table and eccentric load is

|  |  | (S2) |
| --- | --- | --- |

where $a$ is the distance from the center of gravity of the equivalent mass to the center gravity of shaking table, $A$ is the distance from the center of gravity of the load to the center gravity of shaking table.

According to the parallel axis theorem, the $I_{T}$, $I_{TA}$, $I_{L}$, and $I_{LA}$ are

|  |  | (S3) |
| --- | --- | --- |

where $\mathrm{m}$ and $n$ are the length and the width of the shaking table, respectively. $c$ and $k$ are the length and the width of the load, respectively.

According to Eqs. (S1), (S2), and (S3), the moment of inertias in different load conditions are listed in Table S4.

**Table S4.** Moment of inertias in different conditions.

| Conditions | | $I_{T}$($kg\cdot m^{2}$) | $I_{L}$($kg\cdot m^{2}$) | $I_{TA}$ ($kg\cdot m^{2}$) | $I_{LA}$ ($kg\cdot m^{2}$) | $I_{E}$ ($kg\cdot m^{2}$) |
| --- | --- | --- | --- | --- | --- | --- |
| Different MR conditions | 0.5 | $9000$ | $900$ | $153.6$ | $307.2$ | $10360.8$ |
|  | 1.0 |  |  | $345.6$ | $345.6$ | $10591.2$ |
|  | 1.5 |  |  | $497.7$ | $331.8$ | $10729.5$ |
| Different IR conditions | $0.05$ | $9000$ | $450$ | $345.6$ | $345.6$ | $10141.2$ |
|  | $0.1$ |  | $900$ |  |  | $10591.2$ |
|  | $0.15$ |  | $1350$ |  |  | $11041.2$ |
| Different ER conditions | $0.2$ | $9000$ | $900$ | $86.4$ | $86.4$ | $10072.8$ |
|  | $0.4$ |  |  | $345.6$ | $345.6$ | $10591.2$ |
|  | $0.6$ |  |  | $777.6$ | $777.6$ | $11455.2$ |
